# Metabonomic Study on the Plasma of High-Fat Diet-Induced Dyslipidemia Rats Treated with Ge Gen Qin Lian Decoction by Ultrahigh-Performance Liquid Chromatography-Mass Spectrometry

**DOI:** 10.1155/2021/6692456

**Published:** 2021-06-05

**Authors:** Ziwei Xu, Yixuan Sheng, Guowei Zeng, Zhijun Zeng, Bingtao Li, Li Jiang, Guoliang Xu, Qiyun Zhang

**Affiliations:** ^1^School of Pharmacy, Jiangxi University of Jiangxi Chinese Medicine, Nanchang 330004, China; ^2^Jiangxi Province Key Laboratory of TCM Etiopathogenisis, University of Jiangxi Chinese Medicine, Nanchang 330004, China; ^3^Research Center for Differentiation and Development of Basic Theory of TCM, University of Jiangxi Chinese Medicine, Nanchang 330004, China

## Abstract

Gegen Qinlian decoction (GGQLD) has a definite effect on T2DM in clinic, and it has the effect of lowering blood sugar, improving insulin resistance, and improving the blood lipid level of rats with dyslipidemia, but the intervention mechanism of GGQLD on dyslipidemia has not been clarified. The changes in endogenous metabolites in the plasma of high-fat diet-induced dyslipidemia rats treated with Ge Gen Qin Lian Decoction (GGQLD) were studied to elucidate the therapeutic effects and mechanism of action of GGQLD in dyslipidemia. Based on ultrahigh-performance liquid chromatography coupled with quadrupole-time-of-flight tandem mass spectrometry (UHPLC-Q-TOF-MS), the metabolic profiles of rat serum samples were collected. The rat model of dyslipidemia was induced by a 60% fat-fed high-fat diet. After feeding the rats with a high-fat diet for 4 weeks, dyslipidemia appeared. After 5 weeks of GGQLD (14.85 g kg^−1^) administration, the metabonomics of rats' plasma samples in the normal group, model group, and administration group were analyzed. Mass profiler professional (MPP), SIMCA-P 14.1, and Graphpad prism 6.0 software were used combined with METLIN biological database and human metabolite database HMDB to screen and identify endogenous biomarkers. Metaboanalyst 4.0 software was used by combining with HMDB and KEGG databases; the enrichment and metabolic pathway of biomarkers were analyzed to explore the metabolic mechanism of dyslipidemia rats induced by high-fat diet and the intervention mechanism of Gegen Qinlian decoction. After 5 weeks of administration of GGQLD, the levels of serum TC and TG were significantly decreased (*P* < 0.05, *P* < 0.01), while HDL-C and LDL-C were not significantly affected. After administration, the food intake of rats in the administration group decreased gradually, and the change trend of body weight gradually slowed down. The metabonomics of rat plasma samples results showed that 23 potential biomarkers including *α*-linolenic acid, arachidonic acid, and lysophosphatidylcholine were significantly changed in positive ion mode. Studies have shown that GGQLD has a significant lipid-lowering effect on dyslipidemia rats induced by a high-fat diet, and its preventive mechanism is related to tryptophan metabolism, fatty acid biosynthesis, *α*-linolenic acid metabolism, arachidonic acid, and glycerophosphatidyl metabolism pathway.

## 1. Introduction

Type 2 diabetes mellitus (T2DM) accounts for 90% of the total population of diabetes mellitus, but most patients do not have obvious symptoms, especially in the early stage of diabetes mellitus, which is accompanied by varying degrees of overweight or obesity and dyslipidemia. Dyslipidemia refers to the disorder of lipid metabolism in the body and the disorder of plasma lipoprotein exceeding the normal range, accompanied by the increase of total cholesterol (TC), triglyceride (TG), and low-density lipoprotein-cholesterol (LDL-C) and the decrease of high-density lipoprotein-cholesterol (HDL-C). With the popularization of Western fast food, high-fat and high-calorie diet and other bad habits, dyslipidemia and obesity have become a common phenomenon and the incidence rate of dyslipidemia in T2DM patients is 50% [1].

Liu et al. [2] showed that, with the increase of TC and TG levels, the prevalence of T2DM increased significantly. Dyslipidemia is also a risk factor for diabetic complications [3,4]. Dyslipidemia is an important inducement of insulin resistance and plays an important role in the occurrence and development of T2DM [5]. Adiels et al. [6] studied showed that dyslipidemia is an early event of T2DM. The results of the UK Prospective Diabetes Study (UKPDS) show that dyslipidemia is an independent risk factor of T2DM, and there is significant dyslipidemia in the early stage of the disease.

Blood lipid parameters can predict the occurrence of type 2 diabetes to a certain extent. The close attention to the level of blood lipid and timely intervention when abnormal blood lipid occurs are of great significance to delay and control the occurrence and development of type 2 diabetes mellitus and its complications.

Gegen Qinlian Decoction originated from Zhang Zhongjing's Treatise on Typhoid Fever. It is composed of four herbs: *Pueraria lobata* (Willd.) Ohwi (Ge-Gen), *Scutellaria baicalensis* Georgi (Huang-Qin), *Coptis Chinensis* Franch (Huang-Lian), and *Glycyrrhiza uralensis* Fisch (Gan-Cao). As an important herbal medicine in compound prescription, *Pueraria lobata* has been widely used in the treatment of cardiovascular diseases and endocrine and metabolic diseases [7–11], among which dyslipidemia is one of the representatives [12]. Modern pharmacological studies have shown that as berberine was administered to humans and animals over a period of time, the contents of TG, TC, and LDL-C decreased significantly, while the contents of HDL-C increased significantly in hyperlipidemic rats, and berberine can inhibit cholesterol synthesis, promote cholesterol transport and removal, and reduce cholesterol [13–16].

The total flavonoids contained in *Scutellaria baicalensis* have the effect of lowering blood cholesterol. Preliminary clinical studies have shown that the total flavonoids of *Scutellaria baicalensis* have a certain preventive and therapeutic effect on dyslipidemia [17–19].

In summary, the pharmacological studies on the single drug or monomer components in the compound and compound prescriptions suggest that Gegen Qinlian decoction has a certain effect on improving blood lipid.

The preliminary experimental studies of the research group show that Gegen Qinlian decoction has a definite effect on T2DM in clinic, and it has the effect of lowering blood sugar and improving insulin resistance [20–22]. However, the metabolic mechanism and GGQLD intervention mechanism of dyslipidemia rats in the early stage of diabetes have not been clarified.

In recent years, metabonomics has been widely used in the research of disease phenotype, biomarker discovery of drug toxicology, metabolism phenotype, and physiological function of experimental animals, comprehensively depicting the overall characteristics of metabolism [23,24]. In order to study the metabolic mechanism of dyslipidemia and try to find the key biomarkers in the intervention of Gegen Qinlian decoction on dyslipidemia, in this experiment, the model of dyslipidemia was established by feeding rats with a high-fat diet. And the metabolic mechanism of dyslipidemia induced by a high-fat diet was explored; Gegen Qinlian decoction as the intervention drug was used to find the endogenous markers of dyslipidemia rats, and the possible mechanism of Gegen Qinlian Decoction intervention in dyslipidemia rats, which provides a scientific basis to prevent and treat T2DM.

## 2. Materials and Methods

### 2.1. Animals and Ethical Statement

Animal studies were performed according to the Principles of Laboratory Animal Care (World Health Organization, Geneva, 1985). 48 male Sprague–Dawley rats (180 ± 20 g) were purchased from the Jiangxi University of Traditional Chinese Medicine (Certificate: SCXK(Gan)2017-0004, Nanchang, China). This experiment was approved by the experimental animal ethics committee of the Jiangxi University of Chinese Medicine (no. JZSYDWLL-20200828). The rats were housed in a specific pathogen-free breeding room (temperature: 20 ± 2°C; humidity: 60 ± 5%; 12 h light-dark cycle). All of the rats were provided with free access to tap water. Ordinary rat feed was purchased from the Laboratory Animal Science and Technology Center of Jiangxi University of Traditional Chinese Medicine. High-fat feed (D12492): 60% fat, 20% protein, and 20% sugar. The formula is from Research Diets Company (USA).

### 2.2. Instruments

Modular *p*800 Biochemical analyzer (Roche, Germany), high-speed refrigeration centrifuge (Thermo Scientific, Germany), BT225-Electronic analytical balance (Sartorius, Germany), SpeedVac® SPD131Centrifuge enrichment system (Thermo Scientific, Germany), Milli-Q Advantage A10 Ultra-pure Water Purifier (Millipore, USA), Agilent 6538A ultrahigh-performance liquid chromatography-time-of-flight mass spectrometry (UHPLC-Q-TOF-MS) (Agilent, USA).

### 2.3. Reagents

Cholesterol and triglyceride were purchased from Roch (700487-01, Germany), High- and low-density lipoprotein (HL8108 m) and glucose reagent (GL8322]) were obtained from Purebio Biotechnology (Ningbo, China). HPLC-grade formic acid was purchased from Dikma (Richmond Hill, NY, USA). HPLC-grade methanol and acetonitrile were obtained from Tedia (Fairfield, OH, USA). Deionized water was used. The GGQLD formula ratio of radix puerariae, rhizoma coptidis, radix scutellariae, licorice is 8 : 3 : 3 : 2. The decoction preparation method is the same as that of clinical drugs. The added water was 8 times the amount of the decoction pieces, boiled, decocted for 40 min, concentrated to 1 g drug/ml, stored at −20°C for later use, and performed quality control by Agilent 1260 HPLC. The fingerprint of Gegen Qinlian Decoction for this study can be found in the Supplementary Materials (available here).

### 2.4. Establishment of the Rat Model with Dyslipidemia

24 male SD rats were adaptively fed for 5 d, they were randomly divided into the model group (16 rats) and normal group (8 rats) according to body weight. Normal rats were fed with ordinary feed, while model group rats were fed with 60% high-fat feed.

The changes in dietary intake, phenotypic characteristics, and body weight were observed every day, body length was observed, and Lee's index (Lee's index = weight (*g*) ^ (1/3) × 10/body length (mm)) was calculated every week. After four weeks, four blood lipids and serum insulin were measured and IR index was calculated. The blood lipid levels and FPG, Fins, IR index (IR index = fasting blood glucose concentration (mmol/L) × serum insulin concentration (MU/L)/22.5) levels of rats in each group after grouping are shown in Tables 1 and 2.

### 2.5. Animal Model and Drug Administration

The control and dyslipidemia rats were randomly divided into 3 groups: the control group (8 rats treated with saline in a matched volume), dyslipidemia model group (8 dyslipidemia rats treated with saline in a matched volume), and dyslipidemia Gegen Qinlian Decoction group (8 dyslipidemia rats treated with 14.85 g of GGQLD crude drug/kg body weight). GGQLD was orally administered for five consecutive weeks and the model group and the administration group were fed a high-fat diet. After the final administration, the rats fasted for 12 h with free access to water and then were anesthetized with ether, and orbital blood was collected for 1.2 mL to prepare serum and plasma. Blood samples were placed in normal Eppendorf (EP) tubes and EP tubes containing 0.1% heparin sodium, respectively. The blood samples were kept at 4°C for 3 h and centrifuged at 4°C for 15 min (2000 ×g). The supernatant in the normal EP tube was absorbed and stored at 80°C for the detection of biochemical indicators. Meanwhile, the EP tubes contain 0.1% heparin sodium for plasma samples.

### 2.6. Sample Preparation

Frozen plasma samples were thawed at room temperature. Then, 100 *μ*L of the sample was placed in EP tubes, and 300 *μ*L of methanol was added. The tubes were vortexed for 1 min, incubated for 3 h at 4°C, and then centrifuged (21300 ×g, 10 min, 4°C). The supernatants were collected and dried by SpeedVac®, and the residues were reconstituted in 200 *μ*L of methanol: water (15 : 85). Then, the samples were vortexed for 1 min and centrifuged (30000 ×g, 15 min, 4°C). The supernatants were collected and subsequently analyzed following a previously described UHPLC-Q-TOF-MS-based untargeted metabolic profiling strategy [25].

### 2.7. Data Processing and Statistical Analyses

The collected data were extracted by molecular feature extraction with the retention time, m/z value, and the volume for each compound. The data were converted into the .cef format using Profinder B. 06.0 (Agilent Technologies), and they were processed by Mass Profiler Professional (MPP) v12.1 (Agilent Technologies). To compare the metabolite profiles of the three groups, Student's *t*-test and analysis of variance were conducted. Compounds that satisfied *P* < 0.05 and had a fold change ≥2.0 were selected as preliminary potential biomarkers, and unsupervised principal component analysis (PCA) was performed. The CSV format file with peak area value was derived, and partial least squares discriminant analysis (PLS-DA) was carried out with SIMCA-P software. Compounds with VIP > 1 were screened again as potential biomarkers. The potential biomarkers were then returned to original data for matching the different variable compounds in each group. The endogenous biomarkers were identified by comparing the HMDB database with MS/MS information following a previously described method [25]. MetaboAnalyst 3.0 was used to analyze related metabolic pathway.

## 3. Results

### 3.1. Establishment of the Rat Model with Dyslipidemia and Changes of Blood Lipid Level in Rats after 5 Weeks of Gegen Qinlian Decoction Administration

after feeding a 60% fat-fed high-fat diet for 4 weeks, the weight of rats increased significantly, there was a significant difference in Lee's Indices of rats between the model group and the control group (as shown in the Supplementary Table 1), TC and LDL-C increased significantly, HDL-C decreased significantly, and TG increased without statistical difference. A rat model of abnormal lipid metabolism was established. At this time, there was no disturbance of glucose metabolism in rats. After 12 weeks of feeding, TC, TG and LDL-C of rats were significantly higher than those of the normal group, and HDL-C was significantly lower than that of the normal group. High-fat diet had a greater impact on HDL-C of rats.

Five weeks after GGQLD administration, the levels of TC and TG in model group were significantly higher than those in normal group (*P* < 0.01), while HDL-C was significantly lower (*P* < 0.01). Compared with the model group, the levels of TC and TG in the administered group were significantly decreased (*P* < 0.05, *P* < 0.01), as shown in Table 3. Compared with the normal group, the blood sugar in the model group increased significantly (*P* < 0.01) but remained stable in the range of (6.1–7.9 mmol/). Compared with the model group, the administered group significantly prevented the increase of blood sugar (*P* < 0.05, *P* < 0.01), as shown in Table 4.

### 3.2. LC-MS Analysis of Metabolic Profiles of Plasma Samples

**Figure 1:**
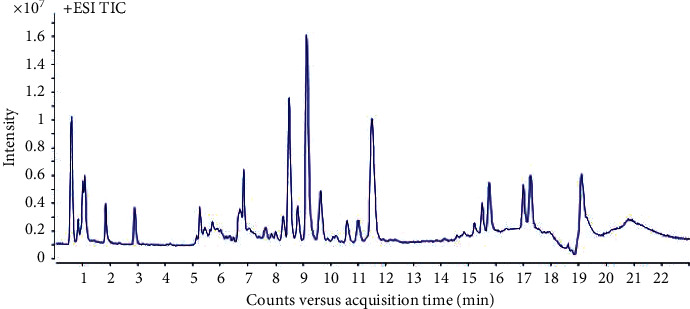
TIC of plasma sample in positive scan mode.

UHPLC-Q-TOF/MS was used to analyze all the plasma samples. LC-MS total ion chromatograms (TIC) of a plasma sample in positive-ion mode is shown in Figure 1. In metabonomics experiments, it is very important to investigate the repeatability of analytical methods because many samples need to be processed.

The quality control (QC) sample was used to monitor the reliability of analytical methods, which was prepared by blending the same volume of liquid from all plasma samples and injecting every five samples to supervise the stability of the analysis in the total run. Relative standard deviations of the retention time and peak area of six selected peaks in quality control samples were <0.17% and <3.58%, respectively (Supplementary Table 2). The resulting data showed that the precision and repeatability of the proposed method were satisfactory for this analysis.

### 3.3. Observation of Principal Component Analysis

**Figure 2:**
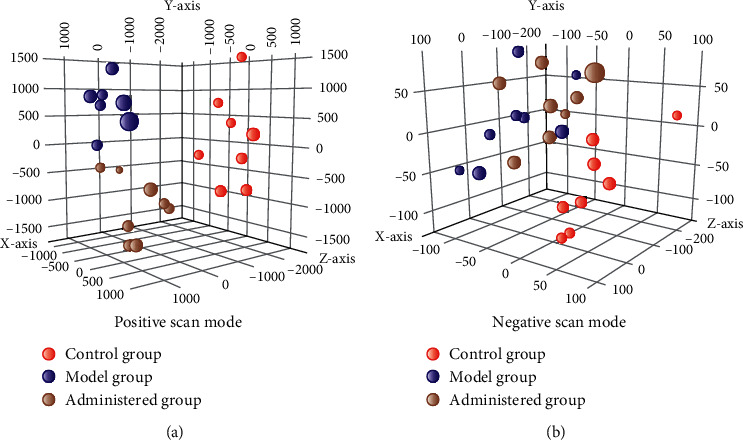
PCA chart of normal group (C), model group (M), and administered group (M).

The endogenous metabolites in plasma samples were detected by UHPLC-Q-TOF/MS in positive full scan mode, to study the effect of Gegen Qinlian Decoction on endogenous substance metabolism in rats with dyslipidemia after administration. The PCA was performed on MPP, and the clustering results of each group of samples are shown in the Figure 2. In the PCA plot, every point represents one plasma sample, and the spatial distribution of the sample represents the metabolic status of different groups of samples.

### 3.4. Partial Least Squares Discriminant Analysis

Compared with unsupervised PCA, supervised partial least squares discriminant analysis (PLS-DA) pays more attention to the compounds contributing to clustering. This experiment further used PLS-DA to screen potential biomarkers.

**Figure 3:**
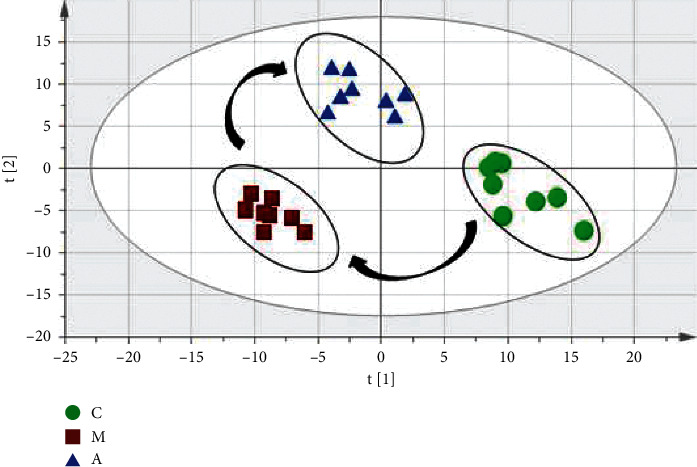
PLS-DA scores of normal group (C), model group (M), and administered group (A).

In this study, PLS-DA was used to analyze the differences among the three groups of samples. The evaluation index R2X of the model indicated the percentage of variation of implicit variable reflecting independent variable X; R2Y indicated the percentage of variation of implicit variable reflecting variable Y; Q2 was the cumulative percentage of the difference between X and Y obtained by the model after cross-validation and predicted the effect of new data. In many cases, the larger the values of R2X, R2Y, and Q2, the closer the ratio of R2Y and Q2 to 1, and the more stable and reliable the model. On the contrary, it means that the model is overfitting. In order to better evaluate the predictability of the model, it is necessary to test whether the reaction of permutation is overfitting. From the score chart (Figure 3), we can see that the normal group (C), model group (M), and Gegen Qinlian decoction administered group (A) were obviously separated (model parameters were R2X = 0.662, R2Y = 0.994, Q2 = 0.903). The normal group (C) and model group (M) were separated along t [1] axis, indicating that the endogenous metabolites of the two groups changed significantly. After treatment with Gegen Qinlian decoction, the serum profile of the administered group moved towards the normal group, indicating that Gegen Qinlian decoction had a reversal effect on the rats with dyslipidemia.

**Figure 4:**
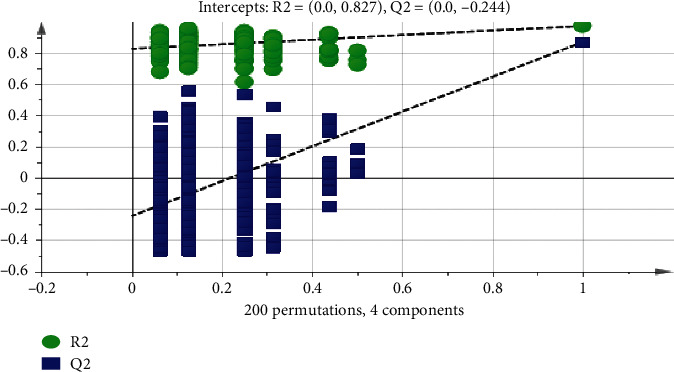
Model verification diagram of three groups.

**Figure 5:**
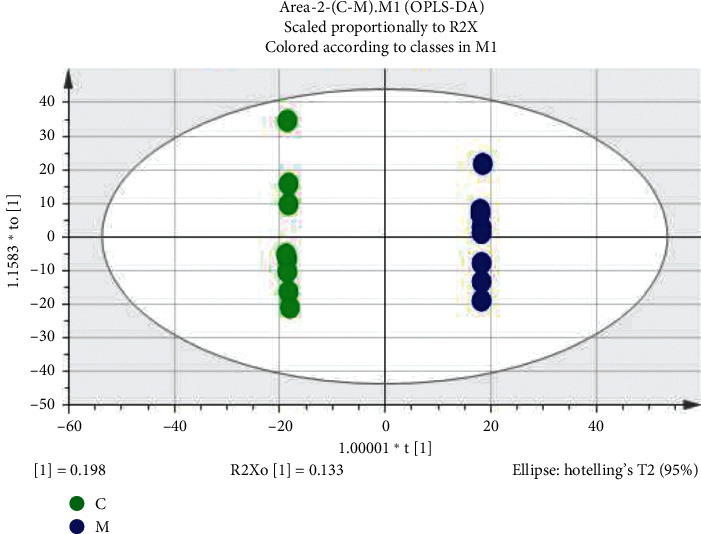
OPLS-DA score map of normal group (C) and model group (M).

**Figure 6:**
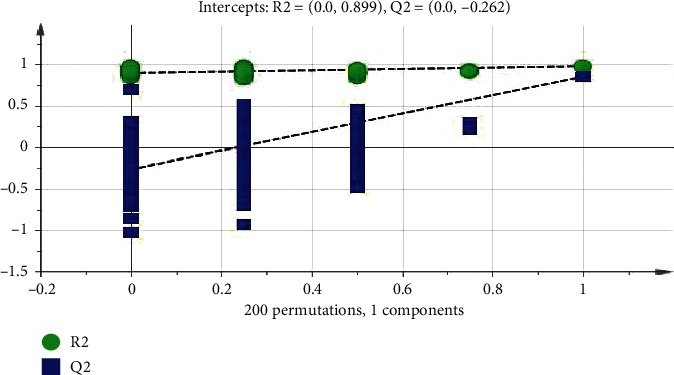
Model verification diagram of normal group (C) and model group (M).

**Figure 7:**
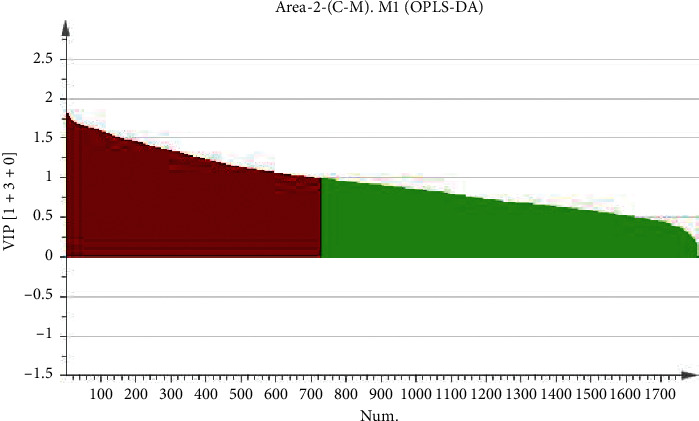
Variable screening chart.

In order to eliminate the overfitting effect of the model, 200 tests were arranged. Generally, the Q2 value on the left side was lower than the original point on the right side, and the intersection point of the vertical axis on the left side and the regression line on the Q2 point was at or below zero. The R2 value on the left side was lower than the original point on the right side, which indicated the validity of the original model.  Figure 4 shows that the model has not been fitted, and the model validation is established. The difference between the normal group and the model group is further analyzed by OPLS-DA. The score chart is shown in Figure 5. The model parameters are R2Y = 0.993, Q2 = 0.873, and the arrangement test is shown in Figure 6. The quality of the model is good. VIP values (the importance of variables in mapping) were extracted from the OPLS-DA model, and the variables with VIP > 1 (Figure 7) were used as the final potential biomarkers for further identification.

### 3.5. Identification of Potential Biomarkers

**Figure 8:**
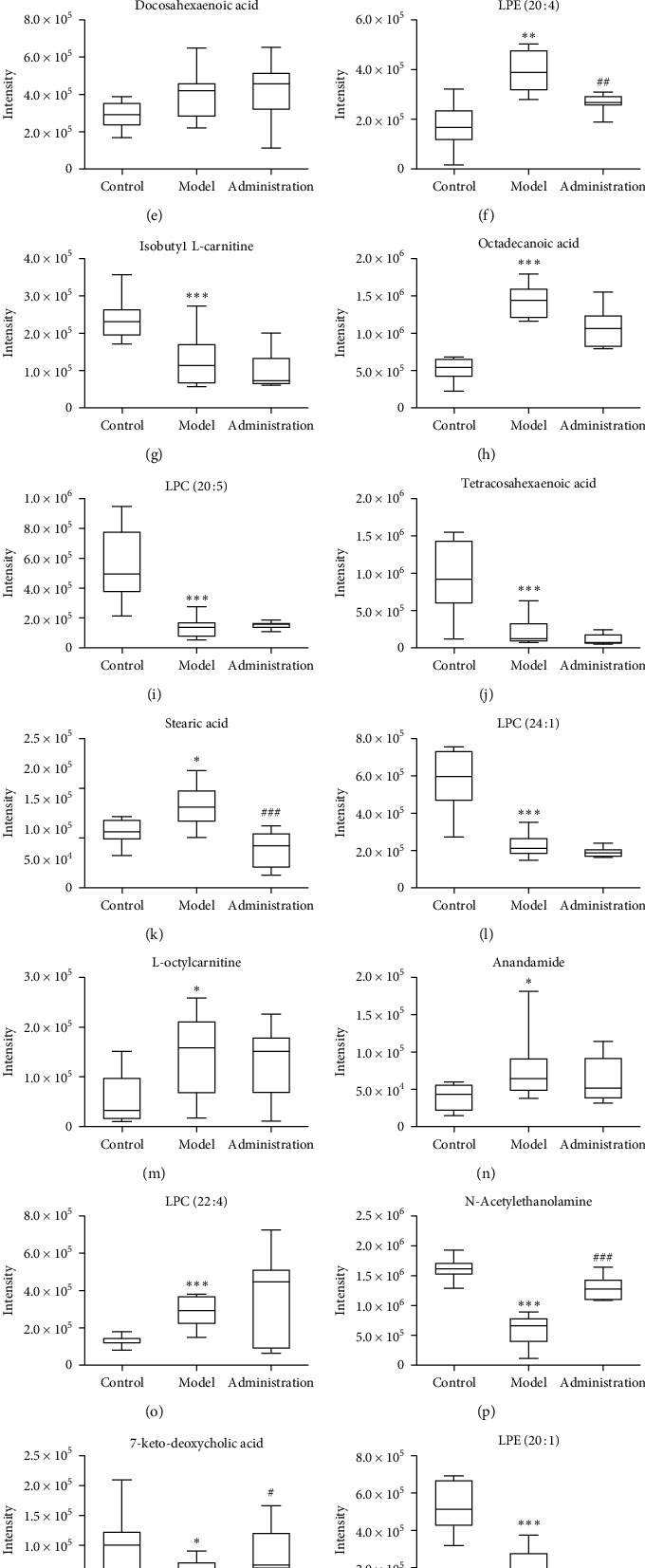
The contents of 19 endogenous compounds in plasma of rats in the control group, model group, and administration group. Note: versus normal group, ^#^*P* < 0.05 and ^##^*P* < 0.01; versus model group, ^*∗*^*P* < 0.05 and ^*∗∗*^*P* < 0.01.

The list of variables (*P* < 0.05, FC (fold change) >2, VIP > 1) was exported to excel, matching the database HMDB with MS/MS information. Mammals- Rattus norvegicus (rat) (KEGG) metabolic pathway analysis showed that potential biomarkers were mainly involved in the biosynthesis of unsaturated fatty acids, *α*-linolenic acid metabolism, glycerophospholipid metabolism, arachidonic acid metabolism, tryptophan metabolism, and fatty acid biosynthesis. In this experiment, the changes of biomarkers in plasma of rats in different groups and involved metabolic pathways were analyzed. The results showed that the contents of some endogenous compounds in rat plasma changed in the model group compared with these of normal rats. After administration of GGQLD, the contents of 16 endogenous compounds (listed in Table 5) in plasma of rats in the administration group returned to normal rats to some extent compared with those in the model group (Figure 8). Through the identification and analysis of 16 biomarkers, the results showed that GGQLD may play a pharmacodynamic role through fatty acid biosynthesis, *α*-linolenic acid metabolism, arachidonic acid metabolism, glycerophospholipid metabolism, tryptophan metabolism pathway.

### 3.6. Metabolic Pathway Analysis

19 potential biomarkers were screened and analyzed by MetaboAnalysis 4.0 combined with pathway-associated metabolite sets (SMPDB) and KEGG for enrichment analysis and pathway analysis.

### 3.7. Enrichment Analysis

**Figure 9:**
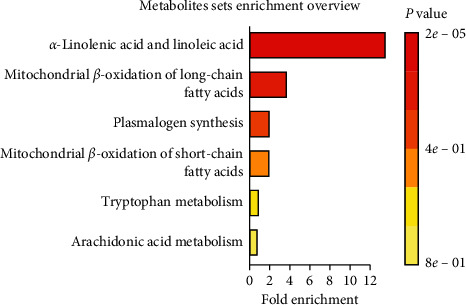
Biomarkers enrichment analysis.

The results of SMPDB enrichment analysis showed that the potential biomarkers were mainly enriched in six metabolic pathways: *α*-linolenic acid and linoleic acid metabolism, long-chain saturated fatty acid mitochondrial beta-oxidation, acetal phospholipid synthesis, short-chain saturated fatty acid mitochondrial beta-oxidation, arachidonic acid metabolism, and tryptophan metabolism (Figure 9, Table 6).

### 3.8. Pathway Analysis

**Figure 10:**
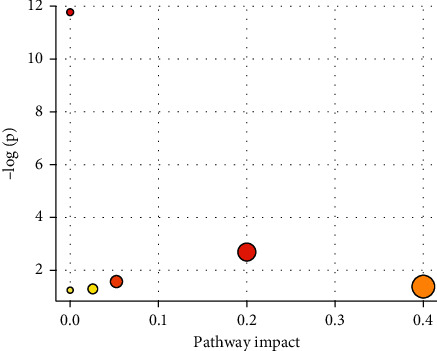
Biomarkers metabolic pathway map.

**Figure 11:**
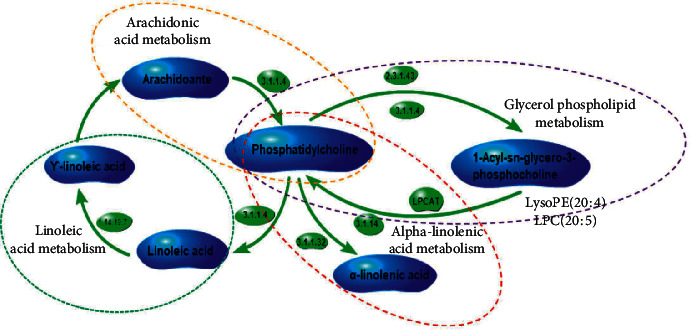
The metabolic pathway network by KEGG.

The results of metabolic pathway analysis by using mammals Rattus norvegicus (rat) (KEGG) showed that the potential biomarkers mainly related to the biosynthesis of unsaturated fatty acids, α-linolenic acid metabolism, glycerophospholipid metabolism, arachidonic acid metabolism, tryptophan metabolism, and fatty acid biosynthesis are shown in Figure 10 and Table 7. The metabolic pathway network mapped by KEGG is shown in Figure 11. Figures 10 and 11 can directly show the metabolic pathways related to the intervention of GGQLD decoction on dyslipidemia.

## 4. Discussion

### 4.1. Establishment of the Rat Model with Dyslipidemia

Abnormal blood lipid is a sign of dyslipidemia. Using 60% high-fat feed for modeling gradually increased the proportion of high-fat feed in normal feed. After modeling, the rats were fed according to body weight. After 4 weeks, TC and LDL-C were significantly increased, while HDL-C was significantly decreased, body weight and Lee's index were significantly increased, and there were no significant differences in fasting blood glucose, serum insulin, and insulin resistance index, indicating that insulin resistance was not caused by dyslipidemia after rats were fed with a high-fat diet.

### 4.2. Biosynthesis of Fatty Acid

In this experiment, compared with the normal group, the contents of *α*-linolenic acid, docosahexaenoic acid, arachidonic acid, octadecanoic acid, docosahexaenoic acid, and stearic acid in the plasma of rats in the model group were significantly changed. After GGQLD was administered, compared with the model group, the contents of the above components in the plasma of the rats in the administration group all returned to the level of the normal group.

The reported study showed that polyunsaturated fatty acids (PUFAs) are important components of cell membrane phospholipids. The synthesis of PUFAs is based on the catalysis, dehydrogenation, and prolongation of stearic acid through a series of enzymes [26]. In mammalian livers, eicosapentaenoic acid is catalyzed by Δ5 and Δ7 lengthening enzymes for two times to form 24-carbon pentaenoic acid, which is catalyzed by Δ6 desaturase to form 24-carbon hexaenoic acid, which is then transferred to the peroxidase body through endoplasmic reticulum for a beta-oxidation to form docosahexaenoic acid. Omega 3 fatty acids such as docosahexaenoic acid can reduce the levels of TC, TG, and LDL-C, which are beneficial to cardiovascular health [27].

The mechanism of the lipid-lowering effect of GGQLD is related to the biosynthesis of fatty acids in vivo; *ω*-3 polyunsaturated fatty acids mainly include *α*-linolenic acid (ALA), eicosapentaenoic acid (EPA), and docosahexaenoic acid (DHA), which have the functions of protecting the cardiovascular system; regulating blood lipid; being anti-inflammation, antiallergy, and antitumor; and improving immune regulation. The effects of *ω*-3 polyunsaturated fatty acids on blood lipids were different in different studies, but the effects on TG were similar. *ω*-3 polyunsaturated fatty acids have a direct inhibitory effect on the synthesis of TG, which may be related to the increase of beta-oxidation and the clearance of TG-rich lipoproteins. ALA, as a maternal body, can be used as a beneficial supplement in diet. It can significantly reduce the TC, TG, LDL-C, and body weight of rats induced by a high-fat diet, increase the level of HDL-C, and prevent and treat hyperlipidemia [28]. ALA may play a role in reducing body weight and blood lipids by accelerating lipid oxidation, affecting the activities of key metabolic enzymes, and reducing visceral fat accumulation [29]. After a high-fat diet, the circulatory alpha-linolenic acid in rats decreased significantly, which increased the risk of cardiovascular disease. After GGQLD intervention, the level of alpha-linolenic acid was significantly reversed, and the concentration of TC and TG was decreased by regulating the metabolism of alpha-linolenic acid in rats.

### 4.3. Arachidonic Acid Metabolism

Arachidonic acid (AA) metabolism is the core of the inflammatory metabolic network. The two main metabolic pathways are the cyclooxygenase (COX) pathway and perlipoxygenase (LOX) pathway. Prostaglandins (PGs), thromboxanes (TXs), leukotrienes (LTs), and lipid peroxides produced by COX pathway are potential targets for anti-inflammatory research [30].

AA metabolic abnormalities are associated with dyslipidemia and coronary heart disease. Thromboxane A2 and prostaglandin I2, metabolites of AA, promote platelet aggregation and agglutination, respectively. Their balance maintains smooth blood circulation and protects endothelial cells from damage. When blood lipid is abnormal, AA synthesizes thromboxane A2 more quickly, which decreases the ability of the blood vessel wall to synthesize prostaglandin I2. It was found that the ratio of plasma lipid peroxide and thromboxane A2/prostaglandin I2 increased in hyperlipidemic rats [31]. Studies have shown that thromboxane A2 and prostaglandin I2 are associated with TC and TG [32,33]. Puerarin can promote vascular endothelial growth factor-like effect and inhibit the increase of thromboxane A2/prostaglandin I2 ratio induced by high-fat diet [34].

In this study, after GGQLD administration, the AA content in plasma of rats in the administration group decreased to the normal group as compared with that in the model group, and its lipid-lowering effect may be one of the mechanisms affecting arachidonic acid metabolism.

### 4.4. Glycerophospholipid Metabolism

Lysophosphatidylcholine is a kind of phosphatidylcholine which contains a fatty acid chain [35]. It mainly refers to lysophosphatidylcholine (LPC), also known as hemolytic lecithin, followed by lysophosphatidylcholine ethanolamine (LPE), which participates in glycerol phospholipid metabolism. LPC is formed by the hydrolysis of phosphatidylcholine by phospholipase A2 or by the hydrolysis of lecithin-cholesterol acyltransferase (LCAT), producing fatty acids such as arachidonic acid, which is closely related to inflammation. LPC plays a role in lipid signaling by acting on LPC receptors, which are members of the G protein-coupled receptor family and participate in many kinds of cell-to-cell signaling. LPC is closely related to metabolic diseases such as dyslipidemia, diabetes mellitus, and cardiovascular diseases [36].

Studies have shown that LPC is the core component of oxidized low-density lipoprotein, which can change endothelial cell permeability and damage endothelial cells [37]. Many studies have reported a significant increase in plasma LPC in obesity or T2DM [38–40]. The study showed that the plasma LPC level significantly decreased after high-fat induction in rats. Regression analysis confirmed that part of LPC was related to IR and that diet and obesity were the main factors affecting blood LPC [39]. LPC is involved not only in cell proliferation, tumor cell invasion, and inflammation but also in glucose metabolism. Yea et al. [41] showed that LPC (16 : 0) and LPC (14 : 0) could stimulate the glucose uptake of 3T3-L1 adipocytes and significantly reduce the blood sugar level of T2DM mice. In this study, after GGQLD administration, the plasma lysophosphatidylcholine level of rats in the administration group was reversed from that of the model group to that of the normal group, possibly through regulating the metabolism of glycerophospholipids to play its hypoglycemic and lipid-lowering role. Its specific mechanism of action and the diverse biological functions of lysophosphatidylcholine deserve further study.

### 4.5. Tryptophan Metabolism

Indoleacetaldehyde (IAALD) belongs to indole derivatives and participates in tryptophan metabolism. Tryptophan (TRP) in humans and animals is mainly brought in by diet, mainly metabolized by kynurenine (KYN), and small amounts of which were metabolized through 5-hydroxytryptamine and indole-retaining pathway.

In this study, IAALD production was significantly reduced in the plasma of model group rats, suggesting that the KYN pathway metabolized by indole 2, 3-dioxygenase (IDO) or tryptophan 2, 3-dioxygenase (TDO) increased, which is related to inflammation and cardiovascular disease. IDO was positively correlated with age and body mass index and negatively correlated with HDL-C. One research [42] showed that blood TRP, KYN, and KYN/TRP ratios are associated with obesity. The abnormal blood lipid and obesity of organisms make it in a chronic low-grade inflammatory state. Proinflammatory cytokines induce the increase of IDO expression, enhance the decomposition of TRP, and increase the production of the toxic metabolites in the KYN pathway. Recently, a prospective study [43] analyzed the correlation between tryptophan metabolism and T2DM, indicating that TRP and its metabolites increased significantly in the early stage of T2DM and decreased in the complete state of T2DM, predicting IR and assessing the risk of T2DM.

In addition, as an essential amino acid, the metabolism of TRP is closely related to intestinal microflora. In the intestine, intestinal microorganisms can convert dietary tryptophan into indole and indole derivatives, including indole propionic acid, indole acetic acid, and indole acetaldehyde. The changes of indole derivatives in vivo after a high-fat diet suggest the imbalance of intestinal microflora. The main components of GGQLD have an anti-inflammatory effect; they can increase the contents of TRP metabolites to a certain extent. GGQLD may play a role in regulating TRP metabolism and intestinal homeostasis by inhibiting the activation of IDO.

### 4.6. Metabolism of Arachidonic Acid Ethanolamine

The endogenous cannabinoid system (ECS) includes two cannabinoid receptors: CB1 and CB2. Two endogenous ligands, arachidonic ethanolamine (AEA) and arachidonic glycerol (2-AG), are also known as endocannabinoids. AEA mainly binds to the CB1 receptor and acts as a partial agonist. The content level of AEA in the blood can reflect the change of ECS in vivo to a certain degree. ECS participates in the regulation of food intake, lipid metabolism, and energy metabolism and plays a direct role in adipogenesis. In the pathological model induced by a high-fat diet, ECS is overactivated, the expression of endogenous cannabinoid is increased, the biosynthesis of fatty acids and triglycerides in the liver is induced, and the differentiation and maturation of preadipocytes are promoted. Adipocytes inhibit fat breakdown and promote obesity. The results showed that the expression of CB1 in peripheral tissues increased in obese groups and animal models induced by a high-fat diet, which eventually led to the disorder of glucose and lipid metabolism [44]. The level of TC and TG could be effectively reduced by giving CB1 antagonists to diet-fattening mice [6]. In this experiment, after 5 weeks of intervention with Gegen Qinlian Decoction, the appetite of rats decreased; the weight gain began to slow down; TC, TG, and FPG decreased significantly; and GGQLD could reduce the level of AEA in plasma of rats with dyslipidemia to a certain extent. It may inhibit appetite by inhibiting the expression of cannabinoid receptors in the central and peripheral nervous system and accelerating the metabolism of peripheral fat, so as to improve the disorder of glycolipid metabolism.

### 4.7. Acyl Carnitines

L-Carnitine lipid is the key substance of lipid metabolism. Fatty acids combine with L-carnitine to form aliphatic carnitine, which passes through the mitochondrial membrane and enters the mitochondrial matrix under the mediation of aliphatic carnitine transferase and then oxidizes and decomposes to release energy. L-carnitine can accelerate fat metabolism; improve heart function; reduce TC and TG; increase HDL-C; and reduce body weight. If the fatty acid is not effectively oxidized by beta, it will cause accumulation of fatty acid, easily produce lipid toxicity, and promote the development of inflammation. The increase of free fatty acids in rats with dyslipidemia and obesity requires more L-carnitine for effective beta-oxidation. The decrease of L-carnitine may not be enough to compensate for the increase of free fatty acids by beta-oxidation, leading to lipid metabolism disorder. Incomplete oxidation-derived acylcarnitine is significantly associated with metabolic diseases, such as long-chain acylcarnitine C18 in diet-induced obese rats. In this study, the plasma levels of L-octyl carnitine and long-chain acylcarnitine stearyl carnitine (C18 : 0) in the model group were significantly higher than those in the normal group, which was consistent with the changes of acylcarnitine metabolic profiles in patients with pre-T2DM [45]. One article showed that the increase of long-chain acylcarnitine in circulation can interfere with insulin signal transduction in cell membranes, which is related to the occurrence and development of insulin resistance, but the exact mechanism of action has not yet been elucidated [46]. Gegen Qinlian Decoction can regulate the level of acylcarnitine to a certain extent, which may promote the beta-oxidation of fatty acids in many ways. Meanwhile, the specific contribution of acylcarnitine of various chain lengths to its biological activity deserves further study.

### 4.8. Sphingolipid and Bile Acid Metabolism

In this experiment, the contents of sphingolipids and bile acids in the plasma of rats in each group were significantly changed. Sphingolipids are an important component of cell membranes and participate in important physiological processes such as cell growth, differentiation, and apoptosis [47]. In obese individuals, sphingolipids accumulate abnormally in tissues and cells, and circulatory levels increase abnormally. Increased sphingolipids can induce insulin resistance by interfering with signal transduction of insulin signaling pathway and promoting cell apoptosis. Bile acids are steroid acids mainly found in mammalian bile. Bile acid can regulate the digestion and absorption of intestinal and liver lipids, and its transformation is closely related to intestinal flora [48]. Bile acid involves key enzymes that balance cholesterol in vivo. Bile acid participates in glycolipid and energy metabolism [49] through different pathways. In this experiment, compared with the normal group, the increase of sphingolipid content in plasma and the decrease of bile acid content in the model group suggest that the obstacle of energy consumption and the accumulation of energy accelerate fat synthesis and promote abnormal blood lipid and weight gain. Studying the changes of sphingolipid metabolism and bile acid levels in organisms has been widely applied to the study of metabolic diseases such as dyslipidemia, obesity, and T2DM and provides new ideas for preventing obesity and metabolic diseases related to obesity.

## 5. Conclusion

Metabolomic studies have shown that a 60% fat-fed high-fat diet involves changes of substances in hemolytic phospholipids, sphingomyelin, bile acid, acylcarnitine, unsaturated long-chain fatty acids, and saturated long-chain fatty acids. Gegen Qinlian Decoction has a significant lipid-lowering effect on dyslipidemia rats induced by a high-fat diet. Its preventive mechanism is related to tryptophan metabolism, fatty acid biosynthesis, alpha-linolenic acid metabolism, arachidonic acid, and glycerophospholipid metabolism pathway [49].

## Figures and Tables

**Table 1:** Blood lipid levels of rats in each group after grouping ($\bar{x}\pm s$).

Group	*n*	Blood lipid levels of rats (mmol/L)			
		TC	TG	HDL-C	LDL-C
Control	8	1.53 ± 0.22	1.26 ± 0.23	0.67 ± 0.11	0.29 ± 0.10
Model	8	1.91 ± 0.44^*∗*^	1.77 ± 1.14	0.50 ± 0.05^*∗∗*^	0.60 ± 0.31^*∗∗*^
Administered	8	1.96 ± 0.14^*∗∗*^	1.21 ± 0.53	0.56 ± 0.06^*∗∗*^	0.86 ± 0.24^*∗∗*^

*Note.* Versus normal group, ^*∗*^*P* < 0.05 and ^*∗∗*^*P* < 0.01.

**Table 2:** FPG, Fins, and IR index levels of rats in each group after grouping ($\bar{x}\pm s$).

Group	*n*	FPG (mmol/L)	Fins (mU/L)	IR index
Control	8	5.04 ± 0.91	21.79 ± 4.78	4.83 ± 1.18
Model	8	5.36 ± 1.73	23.85 ± 4.69	5.58 ± 1.92
Administered	8	5.34 ± 0.95	23.42 ± 2.65	5.60 ± 1.43

**Table 3:** Changes in blood lipid levels of rats in each group after 5 weeks of administration ($\bar{x}\pm s$).

Group	*N*	Blood lipid (mmol/L)			
		TC	TG	HDL-C	LDL-C
Control	8	1.85 ± 0.25	1.02 ± 0.31	0.73 ± 0.11	0.66 ± 0.09
Model	8	2.16 ± 0.29^#^	1.67 ± 0.64^##^	0.58 ± 0.09^##^	0.78 ± 0.29
Administered	8	1.68 ± 0.22^*∗∗*^	0.80 ± 0.19^*∗∗*^	0.52 ± 0.08	0.80 ± 0.21

*Note.* Compared with the control group, ^#^*P* < 0.05 and ^##^*P* < 0.01, compared with the model group, ^*∗*^*P* < 0.05 and ^*∗∗*^*P* < 0.01.

**Table 4:** Changes in blood sugar of rats in each group after 5 weeks of administration ($\bar{x}\pm s$).

Group	*N*	Blood sugar (mmol/L)
Control	8	3.79 ± 1.04
Model	8	5.57 ± 0.91^##^
Administered	8	4.09 ± 0.76^*∗∗*^

*Note.* Compared with the normal group, ^##^*P* < 0.01; compared with the model group, ^*∗*^*P* < 0.05 and ^*∗∗*^*P* < 0.01.

**Table 5:** Potential biomarkers in positive ion mode.

Biomarker	Formula	m/z	RT (min)	VIP	Model group	Administered group
Indole acetaldehyde	C10 H9 N O	160.0743	1.71	1.34	↓##	↑
Acylglycine	C9 H9 N O3	180.0646	4.17	1.65	↓##	↑^*∗∗*^
Iso-L-carnitine	C11 H21 N O4	232.1534	2.39	1.28	↓##	↓
*α*-Linolenic acid	C18 H30 O2	279.2301	14.55	1.62	↓##	↑^*∗*^
(Z)-13-octadecenoic acid	C18 H34 O2	283.2628	17.16	1.64	↑##	↓^*∗*^
Androstenone 3*α*, 17*β*-diol	C15 H28 N6	293.2458	13.51	1.47	↓##	↑
Arachidonic acid	C20 H32 O2	305.2472	15.51	1.24	↑##	↓
Octadecanoic acid	C18 H36 O2	307.2604	16.39	1.06	↑##	↓^*∗∗*^
L-Octyl carnitine	C16 H25 N5	310.1997	5.74	1.74	↑##	↓
Docosapentaenoic acid	C22 H34 O2	331.2622	12.41	1.41	↑#	↑
Arachidonic acid ethanolamine	C22 H37 N O2	348.2889	16.32	1.30	↑#	↓
Ethyl acetate arachidonic acid	C22 H36 O2	355.2614	6.85	1.08	↓##	↑^*∗∗*^
Tetracosahexaenoic acid	C24 H36 O2	357.2768	7.98	1.28	↓##	↓
N-Acetylethanolamine	C24 H36 O3	373.2722	6.81	1.45	↓##	↑^*∗*^
7-Ketodeoxycholic acid	C24 H38 O5	407.2781	6.47	1.32	↓#	↑^*∗*^
Stearyl carnitine	C21 H45 N7 O2	428.3719	9.32	1.60	↑##	↓
LysoPC (14 : 0/0 : 0)	C24 H41 N3 O6	468.307	7.67	1.32	↓#	↓^*∗∗*^
LysoPE (20 : 4 (5Z, 8Z, 11Z, 14Z)/0 : 0)	C25 H44 NO7P	502.2919	8.49	1.60	↑#	↓^*∗∗*^
LysoPE (20 : 1(11Z)/0 : 0)	C27 H45 N3 O6	508.3371	8.72	1.59	↓##	↓
LysoPC (20 : 5 (5Z, 8Z, 11Z, 14Z, 17Z))	C30 H43 N3 O6	542.3227	7.82	1.65	↓##	↑
LysoPC (22 : 4 (7Z, 10Z, 13Z, 16Z))	C32 H49 N3 O6	572.3698	9.89	1.59	↑##	↑
LysoPC (24 : 1(15Z))	C35 H55 N7 O2	628.4311	17.04	1.51	↓##	↓
SM (D18 : 1/14 : 0)	C39 H70 N4 O5	697.5252	19.17	1.67	↑##	↓

*Note.* “↓” means decreased, “↑” means increased; compared with the normal group, ^#^*P* < 0.05 and ^##^*P* < 0.01; compared with model group, ^*∗*^*P* < 0.05 and ^*∗∗*^*P* < 0.01.

**Table 6:** Potential biomarker enrichment analysis table.

Metabolite set	Total	Hits	Expected	*P*	Holm P	FDR
Alpha-linolenic acid linoleic acid metabolism	19	5	0.38	0.002	0.002	0.002
Mitochondrial beta-oxidation of long-chain saturated fatty acids	28	2	0.55	0.10	1.0	1.0
Plasmalogen synthesis	26	1	0.51	0.41	1.0	1.0
Mitochondrial beta-oxidation of short-chain saturated fatty acids	27	1	0.53	0.42	1.0	1.0
Tryptophan metabolism	60	1	1.17	0.71	1.0	1.0
Arachidonic acid metabolism	69	1	1.32	0.76	1.0	1.0

**Table 7:** Analysis table of potential biomarker metabolic pathways.

Pathway name	MatchStatus	*P*	Holm P	Impact
Biosynthesis of unsaturated fatty acids	5/42	<0.001	<0.001	0.0
Alpha-linolenic acid metabolism	1/9	0.07	1.0	0.2
Glycerophospholipid metabolism	1/30	0.21	1.0	0.05
Arachidonic acid metabolism	1/36	0.25	1.0	0.4
Tryptophan metabolism	1/41	0.28	1.0	0.03
Fatty acid biosynthesis	1/43	0.30	1.0	0.0

## Data Availability

The datasets used and analyzed during the current study are available from the corresponding author upon reasonable request.

## Supplementary Materials

Supplementary Materials The fingerprint of Gegen Qinlian Decoction for this study. Supplementary Table 1: Lee's index of rats fed a high-fat diet for four weeks. Table S1: rats were fed a high-fat diet for four weeks $\left(\bar{x}\pm s\right)$. Table S2: the relative standard deviation (RSDs, (%)) of the retention time and the peak area of 6 selected peaks in the quality control samples in positive ESI modes.
