# Supplementary material for: Metabonomic Study on the Plasma of High-Fat Diet-Induced Dyslipidemia Rats Treated with Ge Gen Qin Lian Decoction by Ultrahigh-Performance Liquid Chromatography-Mass Spectrometry
Source: Evid Based Complement Alternat Med. 2021 Jun 5;2021:6692456. doi: 10.1155/2021/6692456 (PMC8203394; doi:10.1155/2021/6692456)
Supplement: Supplementary Materials — The fingerprint of Gegen Qinlian Decoction for this study. Supplementary Table 1: Lee's index of rats fed a high-fat diet for four weeks. Table S1: rats were fed a high-fat diet for four weeks x¯±s. Table S2: the relative standard deviation (RSDs, (%)) of the retention time and the peak area of 6 selected peaks in the quality control samples in positive ESI modes. [file 6692456.f1.zip › 6692456.f1/Supplementary Material.pdf]

Supplementary Material: The fingerprint of Gegen Qinlian Decoction for this study

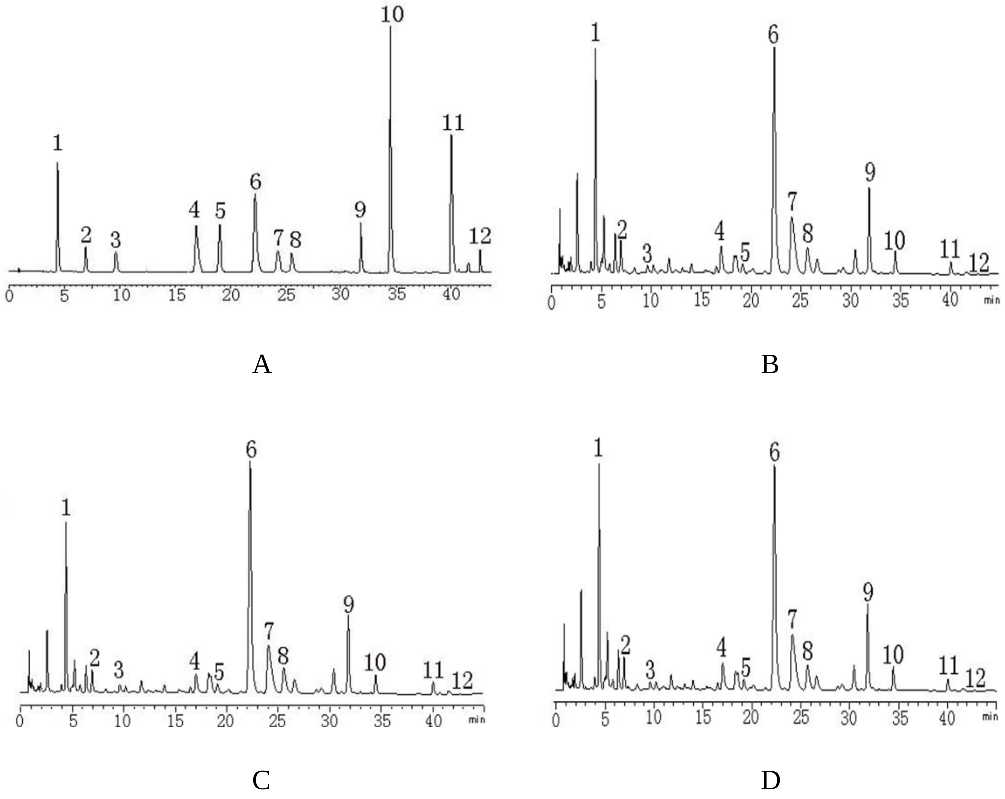

SFig.1. HPLC fingerprint analysis of three batches of GGQLD aqueous preparation. Contents of chemical markers of GGQLD aqueous preparation (Batch A is the sample used in the experiment). (A) HPLC-UV chromatogram of standard reference; (B) HPLC-UV chromatogram of Batch A; (C) HPLC-UV chromatogram of Batch B; (D) HPLC-UV chromatogram of Batch C. Peaks were detected at 275 nm (1, puerarin; 2, daidzin; 3, liquiritin; 4, coptisine; 5, atrorrhizine; 6, baicalin; 7, berberine; 8, palmatine; 9, wogonoside; 10, baicalein; 11, wogonin; 12, ammonium glycyrrhizinate; GGQLD, Gegen Qinlian Decoction.).
