# Supplementary material for: Metabonomic Study on the Plasma of High-Fat Diet-Induced Dyslipidemia Rats Treated with Ge Gen Qin Lian Decoction by Ultrahigh-Performance Liquid Chromatography-Mass Spectrometry
Source: Evid Based Complement Alternat Med. 2021 Jun 5;2021:6692456. doi: 10.1155/2021/6692456 (PMC8203394; doi:10.1155/2021/6692456)
Supplement: Supplementary Materials — The fingerprint of Gegen Qinlian Decoction for this study. Supplementary Table 1: Lee's index of rats fed a high-fat diet for four weeks. Table S1: rats were fed a high-fat diet for four weeks x¯±s. Table S2: the relative standard deviation (RSDs, (%)) of the retention time and the peak area of 6 selected peaks in the quality control samples in positive ESI modes. [file 6692456.f1.zip › 6692456.f1/Supplementary Table 1 (2).pdf]

Supplementary Table 1: The Lee's Index of Rats were fed high fat diet for four weeks

Table S1 Rats were fed high fat diet for four weeks（$\bar{x} \pm s$）

Group	N	Lee's Index
Control	8	294.72 ± 3.19
Model	8	305.59 ± 3.75$^{\#\#}$
Administrated	8	304.10 ± 5.01$^{\#\#}$

Note: Compared with the normal group, $^{\#\#}P < 0.01$
