# Supplementary material for: Metabonomic Study on the Plasma of High-Fat Diet-Induced Dyslipidemia Rats Treated with Ge Gen Qin Lian Decoction by Ultrahigh-Performance Liquid Chromatography-Mass Spectrometry
Source: Evid Based Complement Alternat Med. 2021 Jun 5;2021:6692456. doi: 10.1155/2021/6692456 (PMC8203394; doi:10.1155/2021/6692456)
Supplement: Supplementary Materials — The fingerprint of Gegen Qinlian Decoction for this study. Supplementary Table 1: Lee's index of rats fed a high-fat diet for four weeks. Table S1: rats were fed a high-fat diet for four weeks x¯±s. Table S2: the relative standard deviation (RSDs, (%)) of the retention time and the peak area of 6 selected peaks in the quality control samples in positive ESI modes. [file 6692456.f1.zip › 6692456.f1/Supplementary Table 2.pdf]

Table S2

The relative standard deviation (RSDs,%) of the retention time and the peak area of 6 selected peaks in the quality control samples in positive ESI modes.

Peak no.	m/z value	RT	Peak area	Number
1	190.0851	0.10	2.04	6
2	355.2616	0.07	1.13	6
3	502.2928	0.10	3.58	6
4	373.2723	0.07	1.73	6
5	431.2756	0.10	2.96	6
6	452.1537	0.17	3.26	6
